# Occurrence of neutrophil dysplasia in the course of severe nephrotic syndrome in a 12-year-old boy on immunosuppressive therapy: Questions

**DOI:** 10.1007/s00467-016-3426-1

**Published:** 2016-06-30

**Authors:** Lidia Hyla-Klekot, Paweł Rajwa, Andrzej Paradysz, Piotr Bryniarski

**Affiliations:** 1 Department of Pediatric Nephrology, Pediatrics and Oncology Center, 41-500 Chorzów, Poland; 2 Department of Urology, Medical University of Silesia, 41-800 Zabrze, Poland

**Keywords:** Nephrotic syndrome, Immunosupressive therapy

## Case report

A 12-year-old boy has been treated since he was 9 months old due to severe, recurring, steroid-dependent nephrotic syndrome caused by focal segmental glomerulosclerosis (FSGS). He has been permanently receiving immunosuppressive medications (Table 1).

**Table 1:** Course of the disease and treatment between 2004 and 2014

Years of treatment	Course of the disease	Treatment
2004	Manifestation of the disease, steroid-dependency	GCs, CsA
2004-2006	Relapse of nephrotic syndrome during an infection	CsA, GCs
2008-2009	Two relapses of nephrotic syndrome	GCs, CsA, INN
2010-2011	Remission of nephrotic syndrome	GCs, CsA
2012-2013	Frequent relapses of nephrotic syndrome	GCs, CsA, MMF
2014	Frequent relapses of nephrotic syndrome	CsA (withdrawal), MMF, Rtx

GCs, Glucocorticosteroids; CsA, cyclosporine; INN, cyclophosphamide; MMF, mycophenolate mofetil; Rtx, rituximab

The nephrotic syndrome occurring in the patient can be characterized as follows:

1. The disease developed prior to the boy’s first birthday.
2. Results of genetic tests were negative for mutations of the *nephrin*, *podocin* and *WT1* genes.
3. He has suffered from frequent relapses of nephrotic syndrome.
4. There is evident high-dose steroid dependency that co-exists with cyclosporine A (CsA) toxicity.
5. There is a transient, positive reaction to treatment with alkylating agents.

A renal biopsy performed when the patient was 3 years old revealed a minimal change disease. In 2013, at the age of 10 years, due to an increase in the frequency of relapses the patient was started on combination therapy with CsA and mycophenolate mofetil (MMF). This combination therapy sustained remission for a few months, without any observable clinical side effects or laboratory-proven disorders. Beginning in January 2014 an increase in the frequency of recurrence of nephrotic-range proteinuria was observed, which led to the decision to add methylprednisolone pulses to the therapeutic regimen. A diagnostic biopsy revealed pathological features of FSGS and CsA nephrotoxity. Based on these histopathological findings, the decision was made to discontinue CsA therapy and to administer rituximab (Rtx) with subsequent gradual MMF withdrawal. In August 2014, Rtx was administered in two doses of 375 mg/m^2^ with 1 week between doses. During the period of Rtx administration, the patient was in clinical remission, with proteinuria of <200 mg/24 h, a glomerular filtration rate of 80 ml/min/1.73m^2^ and serum total protein of 6.4 g/dl; no signs of infection were found.

**Fig. 1:**
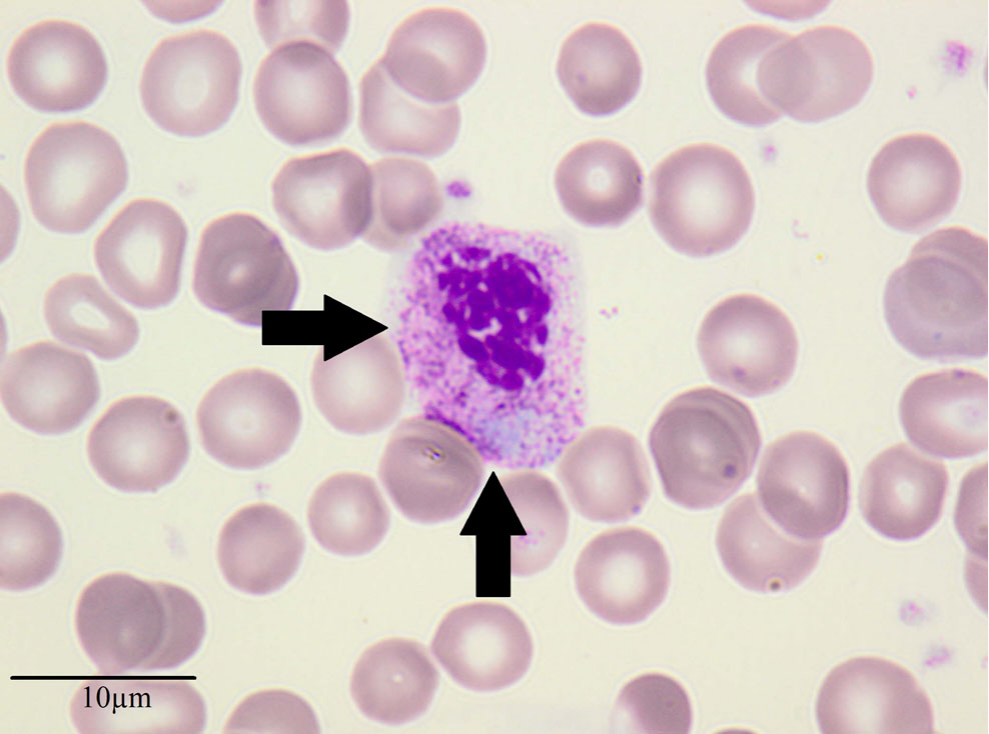
Peripheral blood smear

Directly after the Rtx treatment, the patient received MMF 2 × 1000 mg (650 mg/m^2^) and prednisone (5 mg/day). Flow cytometry at 3 months after the final dose of Rtx revealed total depletion of CD20 B-lymphocytes. Complete blood count showed leukopenia of 2400 cells/cmm with a concomitant increase (in percentage) of immature granulocytes in the peripheral blood (myelocytes 10 %, metamyelocytes 16 %, band cells 7 %, segments 13 %, eosinophils 6 %, basophils 2 %, monocytes 21 %, lymphocytes 47 %). Evaluation of peripheral blood smear revealed the presence of neutrophils with features of dysplasia (asynchronous maturation of the nucleus and the cytoplasm, low nuclear–cytoplasmic ratio, and abundant azurophilic grains with co-existing visible condensations of nuclear chromatin at all stages of development). The number of lobes in mature neutrophils did not exceed three; however, in some of the neutrophils nuclear constriction was not apparent (Fig. 1). Taking into account the long-term immunosuppressive therapy and the increase in the number of immature granulocytes, we decided to establish the differential diagnosis between a myeloproliferative disorder and a reactive process. Reverse transcription-PCR did not detect any BCR-ABL (p210) rearrangement. Bone marrow examination did not confirm the presence of a neoplastic transformation. Virological tests detecting cytomegalovirus and Epstein–Barr virus infection were negative. Owing to the above-mentioned results, we began to suspect that the observed dysgranulopoiesis was associated with the immunosuppressive therapy. In accordance with this suspicion, MMF was withdrawn, which resulted in an immediate normalization of granulocyte morphology and count. During further treatment with prednisone, a depletion of B-lymphocytes was maintained and the 12-year-old boy remained in clinical remission. At the 6-month follow-up we observed an increase in the number of CD20 lymphocytes, which coincided with a relapse of the nephrotic syndrome and symptoms of steroid toxicity and high-dose steroid-dependency.

## Questions

1. What is the final diagnosis of the disorder that was responsible for the neutrophil dysplasia?
2. What does Fig. 1 show?
3. What further treatment may have been effective in the patient?

